# Recent Treatment of Septic Wounds

**Published:** 1916-04

**Authors:** 


**Recent Treatment of Septic Wounds.** A. E. Morrison and W. J. Tulloch, Brit. Jour. of Surg., p. 276, 1915, vol. 3, use sterilized solutions of magnesium sulphate 4 parts, glycerin 1 part, boiling water 3 parts, claiming lack of pain and pus, healthy granulations and the necessity of renewing dressings only twice a day. W. St. C. Symmers and T. S. Kirk, Lancet, Dec. 4, use urea in powder, claiming better results in sloughing infected wounds than from any other method, renewing the application every 24 hours and recommending it as a first aid dressing.

